# Visits to Private Asylums

**Published:** 1903-01-10

**Authors:** 


### VISITS TO PRIVATE ASYLUMS.

By Our Rambling Reporter.

### HEIGHAM HALL, NORWICH.

Norwich is a fine old city, now containing upwards of a hundred thousand inhabitants. On the outskirts of the town, reached by electric tramcar, and not far from the old palace of Bishop Hall, which bears date 1615, stands Heigham Hall. The building is plain, which seems right and proper considering the use to which it is put. It is of various ages, some parts are in Georgian style, and the grounds, about twelve acres in extent, contain an abundance of fine old trees which naturally accentuate the country mansion appearance of the place. The entrance gates stand wide open, the grounds are tastefully laid out, and some of the patients wander about in perfect freedom. Others are allowed to go into the city unattended.

For the most part the rooms are very well furnished, and have a bright, cheerful look, some being made more so by a profusion of flowers. In others, well-filled bookcases are seen. There are a billiard-room, a conservatory, and a large summer-house, all for the use of the patients. The amusements consist in the usual indoor and outdoor games, and country drives are often given. In spite of its 95 beds, there is a general air of homeliness about the asylum, and some of the better patients dine at the proprietor's table. Indeed, except for a locked door now and then met with, and a padded room or two, there is little difference between Heigham Hall and a private house, apart from the presence of the patients. Of course this remark is more or less applicable to all our English licensed houses. We venture to hope that locked doors, by day at all events, will soon be practically abolished in all our private asylums. This open-door system has been found practicable in the public asylums of Scotland, and should therefore be easily engrafted on our private asylums, where the attendants and nurses are relatively twice as numerous.

Heigham Hall has been licensed for about sixty years, and the present licensee is Mr. Alfred Mottram. Dr. McWilliam is the medical superintendent, and there is also an assistant medical officer. The asylum is therefore well officered, and the staff of attendants and nurses is good—one might even say liberal. About 50 per cent. of the patients recover and are discharged, and the deaths would seem to be under 5 per cent. of the average number resident. No mechanical restraint has been used for 15 years, and seclusion is but little resorted to. Unless for some medical reason, patients are allowed to see their friends at any time. The rector of the parish has a service every Sunday in one of the large sitting-rooms. Payments for patients range from £2 2s. to £6 6s. a week.

